# Serum bilirubin level is a strong predictor for disability in activities in daily living (ADL) in Japanese elderly patients with diabetes

**DOI:** 10.1038/s41598-019-43543-6

**Published:** 2019-05-08

**Authors:** Toyoshi Inoguchi, Saki Fukuhara, Mayumi Yamato, Michikazu Nakai, Tomoaki Etoh, Mitsunori Masakado, Satoshi Suehiro, Fumio Umeda, Teruaki Yamauchi

**Affiliations:** 1 Fukuoka City Health Promotion Support Center, Fukuoka, Japan; 2 Yukuhashi Central Hospital, Yukuhashi, Japan; 3 Physical Chemistry for Life Science Laboratory, Faculty of Pharmaceutical Sciences, Kyushu University, Fukuoka, Japan; 4 Department of Statistics and Data Analysis, National Cerebral and Cardiovascular Center, Suita, Osaka Japan

**Keywords:** Predictive markers, Geriatrics

## Abstract

Elderly patients with diabetes are at increased risk of frailty and disability in activities of daily living (ADL). Recent evidence has shown that oxidative stress is associated with these conditions. In this cross-sectional study, we aimed to assess whether serum level of bilirubin, a strong endogenous antioxidant, can predict ADL disability in elderly patients with diabetes. Forty elderly patients aged 70 years and older with diabetes and ADL disability and 158 elderly patients with diabetes and without ADL disability were continuously recruited. Multivariate logistic regression models showed that serum bilirubin level was a significant predictor for ADL disability. Receiver operating characteristic analysis showed that the area under the curve (AUC) of serum bilirubin level alone for ADL disability was 0.887 (95% CI 0.837–0.936, P < 0.001) and the cut-off value was 0.4 mg/dL (sensitivity = 88.0% and specificity = 65.0%). The predictive ability was further increased by the addition of age (AUC = 0.921) or addition of age, body mass index, red blood cell count, cerebrovascular disease and chronic renal failure (AUC = 0.953). In conclusion, low serum bilirubin level is a strong predictive biomarker for ADL disability in elderly patients with diabetes, and its clinical utility is suggested.

## Introduction

Over the last few decades, life expectancy has gradually increased, along with an increase in the incidence of age-related disabilities in activities of daily living (ADL). In addition, diabetes and related complications are significant healthcare problems in the growing elderly population. Recent evidence has indicated that elderly people with diabetes are at increased risk of frailty and ADL disability^1–4^. The identification of potentially treatable risk factors and predictable markers for ADL disability is critical for preventing the onset of frailty and ADL disability in the elderly population, especially in those with diabetes.

ADL disability is an adverse outcome of frailty. Recently, oxidative stress has gained attention as one of the important causative factors of frailty, and increased levels of oxidative stress and proinflammatory biomarkers were observed in physically frail and prefrail subjects^5–7^.

Bilirubin is a tetrapyrrolic compound. The superfamily of tetrapyrrolic compounds are highly conserved and originally serve as light harvesting and energy generating pigments in a photosynthetic process in algae and plants. In human, bilirubin has been recognized as a strong endogenous antioxidant^8^ and thus increasing attention has been recently paid to its protective effects against oxidative stress-induced damage^9,10^. Bilirubin is almost 20 times more effective at preventing low density lipoprotein oxidation compared with vitamin E analogue^9^. More importantly, serum bilirubin plays a main role in the antioxidant capacity in blood^10^. Accumulating clinical evidence has shown the inverse association of serum bilirubin level with the onset and development of oxidative stress-related diseases including diabetes, diabetic vascular complications, chronic kidney disease (CKD) and atherosclerotic diseases^11–16^. Therefore, the objective of this study was to assess the predictive value of serum bilirubin level for ADL disability in elderly patients with diabetes aged 70 years and older.

## Results

**Figure 1:**
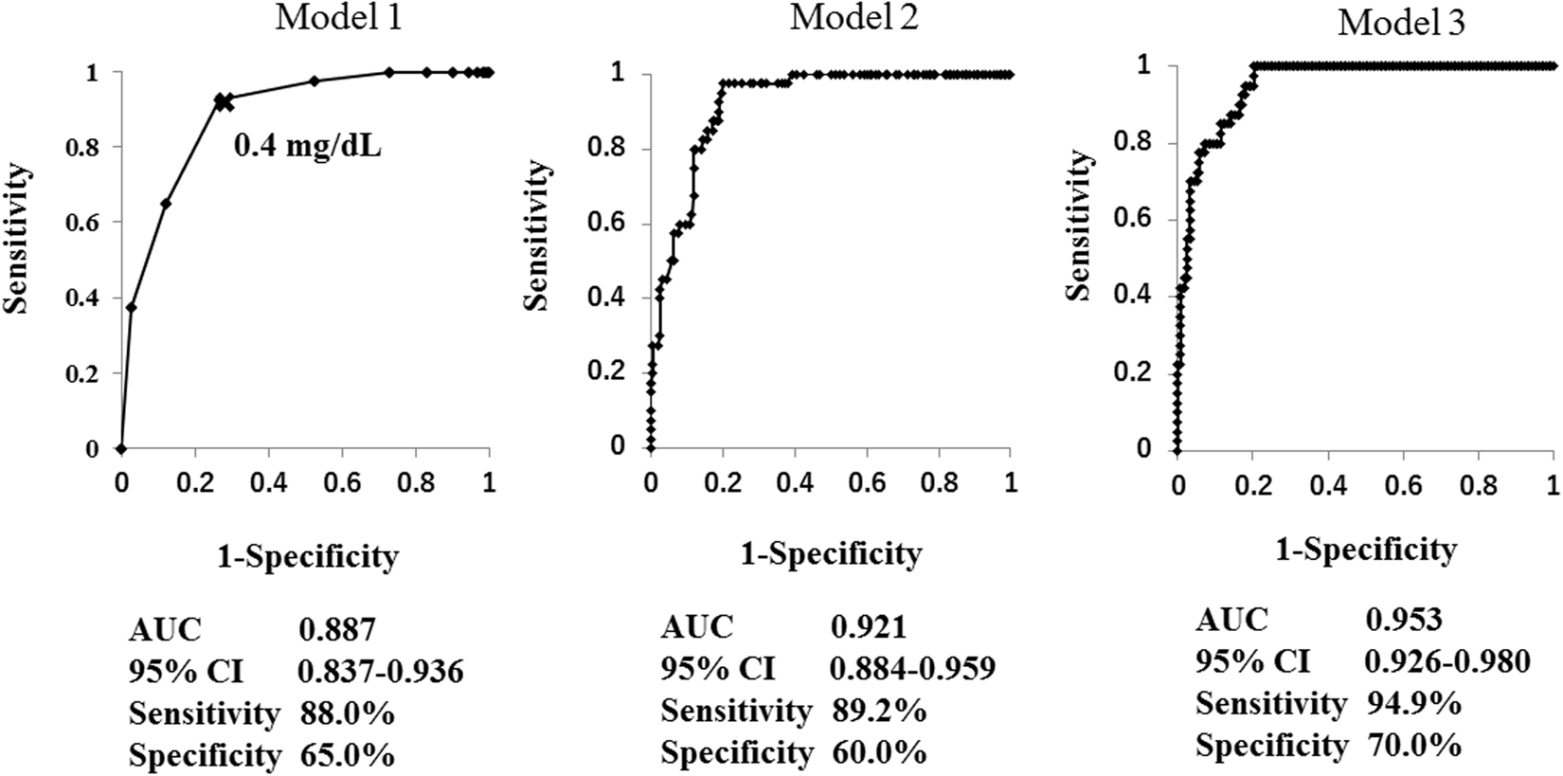
AUC of various models to predict ADL disability evaluated by ROC curves. ROC curves demonstrate the relative ability of serum bilirubin level (Model 1), serum bilirubin level and age (Model 2) and serum bilirubin level, age, body mass index, red blood cell count, cerebrovascular disease and chronic renal failure (Model 3) to predict ADL disability. AUC, area under the curve. ROC, receiver operating characteristic.

Patient characteristics are shown in Table 1. In patients with ADL disability, age and prevalence of chronic renal failure, cerebrovascular disease and proliferative retinopathy were significantly higher, while serum bilirubin level, haemoglobin A1c (HbA1c) level, body mass index (BMI), red blood cell (RBC) count and prevalence of hyperlipidaemia were significantly lower than in those without ADL disability (Table 1). Multivariate logistic regression analyses using various models were performed to assess the predictive value of serum bilirubin level for ADL disability. As shown in Table 2, Model 1 using serum bilirubin level only showed that it was a significant predictive factor for ADL disability (OR 0.293, 95% CI 0.197–0.435, P < 0.001). Model 2 using serum bilirubin level and age showed that both variables were significant predictive factors for ADL disability (OR 0.254, 95% C 0.158–0.409, P < 0.001 and OR 1.203, 95% CI 1.093–1.323, P < 0.001, respectively). Model 3 showed that serum bilirubin level, age, BMI and chronic renal failure were significant predictive factors for ADL disability (OR 0.383, 95% CI 0.226–0.652, P < 0.001; OR 1.194, 95% CI 1.066–1.338, P = 0.002; OR 0.861, 95% CI 0.755–0.983, P = 0.027; and OR 7.931, 95% CI 1.904–33.040, P = 0.005, respectively). Receiver operating characteristic (ROC) analysis was subsequently performed to evaluate the relative ability of each model to predict ADL disability. As shown in Fig. 1, the area under the curve (AUC) in Model 1 was 0.887 (95% CI 0.837–0.936, sensitivity = 88.0%, specificity = 65.0%), and the cut-off value of serum bilirubin level for ADL disability was 0.4 mg/dL. AUC in Model 2 was 0.921 (95% CI 0.884–0.959, sensitivity = 89.2%, specificity = 60.0%, P < 0.001 vs. Model 1), and AUC in Model 3 was 0.953 (95% CI 0.926–0.980, sensitivity = 94.9%, specificity = 70.0%, P < 0.001 vs. Model 2). We also examined the AUC of various variables individually (Table 3). Bilirubin alone had high predictive ability for ADL disability compared with the other variables, although its predictive ability was further increased by the addition of age or factors including age, BMI, RBC count, cerebrovascular disease and chronic renal failure. There was no gender difference in the significant correlation between bilirubin level and disability.

**Table 1:** Comparison of metabolic variables and presence of comorbid diseases between patients with and without ADL disability.

Characteristic	Disability+ n = 40	Disability− n = 158	*P* Value
Sex			
M (%)	11 (27.5)	68 (43.0)	0.073^a^
F (%)	29 (72.5)	90 (57.0)	
Age, median (IQR), yrs	83.0 (78.8–86.0)	76.5 (74.0–81.0)	<0.001^b^
Body mass index, mean (SD)	20.7 (4.8)	23.8 (3.6)	<0.001^c^
HbA1c, median (IQR), %	6.0 (5.6–7.0)	6.9 (6.4–7.5)	0.005^b^
Bilirubin, median (IQR), mg/dL	0.3 (0.2–0.4)	0.5 (0.4–0.7)	<0.001^b^
RBC count (x10^12^/L) (SD)	3.54 (0.65)	4.28 (0.52)	<0.001^c^
Hypertension			
**− (%)**	7 (17.5)	(31.6)	0.078^a^
**+ (%)**	33 (82.5)	141 (68.4)	
Hyperlipidemia			
**− (%)**	18 (45.0)	56 (35.4)	<0.001^a^
**+ (%)**	22 (55.0)	102 (64.6)	
Coronary artery disease			
**− (%)**	27 (67.5)	(74.7)	0.238^a^
**+ (%)**	13 (32.5)	50 (25.3)	
Cerebrovascular disease			
**− (%)**	28 (30.0)	(91.8)	<0.001^a^
**+ (%)**	12 (70.0)	13 (8.2)	
Retinopathy			
− (%)	7 (21.2)	101 (76.4)	<0.001^a^
Simple (%)	2 (6.1)	13 (10.6)	0.2177^a^
Proliferative (%)	24 (72.7)	16 (13.0)	<0.001^a^
Nephropathy			
− (%)	18 (45.0)	132 (83.5)	<0.001^a^
Proteinuria (%)	1 (2.5)	17 (10.8)	0.052^a^
Chronic renal failure (%)	21 (52.5)	9 (5.7)	<0.001^a^

^a^Calculated using the chi-squared test. ^b^Calculated using the Mann–Whitney U test.

^c^Calculated using the t test. Disability+, patients with ADL disability; Disability−, patients without ADL disability.

**Table 2:** Multivariate logistic regression models to predict ADL disability.

Variables	Mode1 1		Model 2		Model 3	
	OR 95% CI	P value	OR 95% CI	P value	OR 95% CI	P value
Serum bilirubin level	0.293	<0.001	0.254	<0.001	0.383	<0.001
(0.1 mg/dL)	0.197–0.435		0.158–0.409		0.226–0.652	
Age (years)			1.203	<0.001	1.194	0.002
			1.093−1.323		1.066–1.338	
Body mass index					0.861	0.027
(m/kg^2^)					0.755–0.983	
Red blood cell count					0.993	0.212
(x10^12^/L)					0.983–1.004	
Cerebrovascular					1.699	0.446
disease					0.435–6.641	
Chronic					7.931	0.005
renal failure					1.904–33.040	

Data are presented as OR and 95% CI. OR represents the increase in odds of ADL disability for every unit increase in the dependent continuous variable or the presence of an independent categorical variable. Model 1: serum bilirubin alone. Model 2: serum bilirubin and age. Model 3: serum bilirubin level, age, body mass index, red blood cell count, cerebrovascular disease and chronic renal failure.

**Table 3:** AUC of individual variables to predict ADL disability evaluated by ROC curves.

Variables	AUC
Age	0.743
Sex	0.577
Body mass index	0.731
HbA1c	0.649
Red blood cell count	0.807
Hypertension	0.571
Hyperlipidemia	0.598
Coronary artery disease	0.545
Cerebrovascular disease	0.609
Proliferative retinopathy	0.799
Chronic renal failure	0.734
Serum bilirubin level	0.887
Serum bilirubin level plus age	0.921
Serum bilirubin level plus 4 variables*	0.953

*Variables include age, body mass index, cerebrovascular disease and nephropathy (chronic renal failure). AUC, area under the curve.

In turn, multivariate regression analysis using stepwise methods showed that ADL disability, chronic renal failure, age and coronary artery disease were significant determinant factors for serum bilirubin level (P = 0.007, P = 0.020, P = 0.043 and P = 0.05, respectively), with standardized partial regression coefficients of −0.236 (95% CI −0.239–0.039), −0.198 (95% CI −0.233–0.020), −0.152 (95% CI −0.013–0.000) and −0.136 (95% CI −0.150–0.000), respectively (Table 4). These results suggest that the presence of ADL disability was most strongly associated with serum bilirubin level.

**Table 4:** Evaluation of significant determinants for serum bilirubin level evaluated by multivariate regression analysis using the stepwise method.

Variables	Partial regression coefficient	S.E.	Standardized partial regression coefficient	95% CI Lower Upper	P value
ADL disability	−0.139	0.051	−0.236	−0.239 −0.039	0.007
Age	−0.007	0.003	−0.152	−0.013 −0.000	0.043
Red blood cell count	0.001	0.000	0.155	+0.001 +0.006	0.063
Coronary artery disease	−0.075	0.038	−0.136	−0.150 −0.000	0.050
Chronic renal failure	−0.127	0.054	−0.198	−0.233 −0.020	0.020

Data are presented as partial regression coefficient and 95% CI.

## Discussion

To our knowledge, this is the first report to show that serum bilirubin level may be a significant predictor for ADL disability in elderly patients with diabetes aged 70 years and older. The cut-off value of serum bilirubin level for ADL disability was 0.4 mg/dL as evaluated by ROC curve. Serum bilirubin level alone had a strong predictive ability for ADL disability with AUC = 0.887 (sensitivity = 88.0%, specificity = 65.0%), although its predictive ability was further increased by the addition of age (AUC = 0.921) or by the addition of factors including age, BMI, RBC count, cerebrovascular disease and chronic renal failure (AUC = 0.953). Notably, previous report showed that serum bilirubin level is inversely associated with functional dependence including ADL in the elderly subjects aged 60 years and older^17^. Since elderly subjects with diabetes are at increased risk of frailty and ADL disability^1–4^, it is clinically important to evaluate the predictive value of serum bilirubin level on ADL disability in those subjects. The present findings further suggested that low serum bilirubin level is a strong biomarker to predict ADL disability in elderly subjects with diabetes.

Bilirubin is a strong endogenous antioxidant^8^. Accumulating clinical and experimental evidence has indicated that bilirubin has a protective effect on various oxidative stress-related diseases including diabetes, diabetic vascular complications, CKD and atherosclerotic diseases^11–16^. We showed for the first time a lower prevalence of vascular complications as well as decreased markers of oxidative stress and inflammation in diabetic patients with Gilbert syndrome, a congenital hyperbilirubinemia^11^. This finding suggests that sustained hyperbilirubinemia may protect against oxidative stress and inflammation, and thus prevent the development of diabetic vascular complications. The beneficial effect of bilirubin has been supported by an a growing body of both clinical and experimental studies^12,13,18^. A recent report showed that serum bilirubin levels were inversely associated with progression of nephropathy in a post hoc analysis of the Reduction of Endpoints in NIDDM with the Angiotensin II Antagonist Losartan (RENAAL) trial^12^. These results were independently confirmed in the Irbesartan Diabetic Nephropathy Trial^12^. A meta-analysis including a total of 132,240 subjects from 27 studies has also shown a significantly negative association between serum bilirubin level and risk of diabetic complications^13^. In rodents, we showed that bilirubin and biliverdin protected against the development of albuminuria and renal mesangial expansion in diabetes, along with inhibition of oxidative stress markers and increased expression of NAD(P)H oxidase^18^. Taken together, these findings suggest that low serum bilirubin level may cause decreased antioxidant activity, subsequently leading to various oxidative stress-related diseases such as diabetic vascular complications. Conversely, recent studies have implicated increased levels of oxidative stress and proinflammatory biomarkers in physically frail and prefrail subjects^5–7^. Sarcopenia is the main domain of these conditions. Although multiple factors are involved in sarcopenia, skeletal muscles consume large quantities of oxygen and generate substantial concentrations of reactive oxygen species (ROS), whose accumulation is thought to cause loss of muscle quantity and quality through several mechanisms^19^. The age-induced decline in Type II muscle fibres may be at least partially attributable to oxidative injury and apoptosis^20^. Increased ROS production may activate the ubiquitin-proteasome system and muscle proteases (caspases, calpains), leading to protein degradation^21^, and also may be associated with intracellular functional changes in fibre activation^21^. Thus, the association between low serum bilirubin level and ADL disability may be due to decreased antioxidative activity or increased oxidative stress. However, this hypothesis should be verified in future studies.

There are several limitations associated with this study. Firstly, this study was not carried out at multiple institutes and the sample size was small. Therefore, we could not completely exclude selection bias of participants, although participants were continuously selected during a fixed period of time. Secondly, the lack of data on antioxidant capacity or oxidative stress markers in serum or the lack of data for unconjugated and conjugated bilirubin level raises the possibility of unmeasured confounders and weakened the results of this study. Thirdly, the most important limitation was that this study was cross-sectional.

In conclusion, the present study showed for the first time that low serum bilirubin level is a strong predictive biomarker for ADL disability in elderly patients with diabetes, and its cut-off value is 0.4 mg/dL. The clinical utility of serum bilirubin level is suggested. The underlying mechanism remains to be clarified in future studies, and prospective and large-scale studies should be carried out to confirm the predictive value of serum bilirubin level.

## Methods

### Participants

Forty elderly patients aged 70 years and older with diabetes and ADL disability under medical care at Yukuhashi Central Hospital from November 2017 to February 2018 were recruited, and 158 elderly patients with diabetes and without ADL disability were continuously recruited. Patients who suffered from hepatobiliary diseases with abnormal levels of aspartate aminotransferase, alanine aminotransferase or alkaline phosphatase and haemolytic anaemia were excluded. All patients classified as having ADL disability in this study had a “Certification of Needed Long-Term Care” by the municipal government. In addition, ADL was evaluated by self-report regarding physical ability in basic self-care tasks (e.g. mobility, bathing, dressing, eating and using the toilet), which was ascertained in an interview by expert nurses. Mobility includes the ability to move one’s own body, such as walking, standing up, turning over in bed and climbing stairs. ADL disability in this study was defined as having disability in two or more tasks.

### Clinical variables and definition

In this study, peripheral venous blood samples were collected after overnight fasting. Serum bilirubin level was measured by the vanadate oxidation method. Total cholesterol, triglyceride and high-density lipoprotein cholesterol levels were measured using standard methods. Low-density lipoprotein (LDL) cholesterol level was calculated using the Friedewald formula. The haemoglobin A1c (HbA1c) value was determined using a standard high-performance liquid chromatography method. Hypertension was defined as systolic blood pressure >140 mmHg or diastolic blood pressure >90 mmHg, or the current use of any antihypertensive medication. Hyperlipidaemia was defined as serum concentration of LDL cholesterol >120 mg/dL and triglyceride >150 mg/dL according to the Japan Diabetes Society criteria, or the current use of lipid-lowering agents. Proteinuria was defined as a urinary albumin to creatinine ratio of >300 mg/g creatinine or a level of + or more using the Albustix (Ames Co., USA) method. Chronic renal failure was defined as estimated glomerular filtration rate (eGFR) < 30 mL/min/1.73 m^2^ or receiving haemodialysis. Retinopathy was assessed by a fundus examination by independent ophthalmologists. Coronary artery disease was defined as a history of acute myocardial infarction or angina pectoris confirmed by clinically significant obstruction on coronary angiography or revascularization with angioplasty or coronary artery bypass. Cerebrovascular disease was defined as a history of symptomatic stroke confirmed by brain computed tomography or magnetic resonance imaging.

### Statistical analysis

A two-sided P value of less than 0.05 was considered significant. Data are presented as mean ± standard deviation (SD) for variables with normal distribution and as median (interquartile range) for variables with non-normal distribution. The significance of differences was determined by the chi-squared test for categorical variables and the unpaired t test or the Mann–Whitney U test for continuous variables. Multivariate logistic regression analysis using various variables was performed to evaluate the predictive value of serum bilirubin level for ADL disability. In this study, we selected serum bilirubin alone for Model 1 and serum bilirubin level and age for Model 2. For Model 3, we selected serum bilirubin level, age, BMI, RBC count and presence of cerebrovascular disease and chronic renal failure because these valuables were significantly correlated with ADL disability in univariate analyses and thought to be clinically important. Proliferative nephropathy was strongly correlated with chronic nephropathy, thus, we selected only the latter. Other variables would not appreciably improve prediction and consequently were not included in the model. Then, we plotted the ROC curve and calculated the AUC, sensitivity and specificity in each model. The optimal cut-off value of bilirubin level for ADL disability was obtained from the Youden index [maximum = sensitivity + specificity − 1]. In turn, to evaluate which variables were significant determinants for serum bilirubin level, multivariate regression analysis was performed. In this analysis, we included all variables in the model and used stepwise methods. We used the Power and Sample Size calculation software version 3.1.2 (biostat.mc.vanderbilt.edu/wiki/Main/PowerSampleSize) to calculate sample size. We had a power of at least 90% with a two-sided α level of 0.05, assuming that the ratio of the number of patients with ADL disability to those without it is 1:4. Statistical analysis was performed using BellCurve for Excel version 2.15 (Tokyo, Japan).

### Ethical approval

All procedures were performed in accordance with the relevant guidelines and regulations. Informed consent was obtained from all participants or their legal guardian. The study was approved by the ethics committee of Yukuhashi Central Hospital (No. 20181126003).

## Data Availability

The datasets generated during and/or analysed during the current study are available from the corresponding author on reasonable request.
